# A Lecture on Tetanus, Delivered at the Philadelphia Medical Institute

**Published:** 1838-09-26

**Authors:** Thomas Harris


### A LECTURE ON TETANUS, *delivered at the Philadelphia Medical Institute, by* THOMAS HARRIS, M. D.

TETANUS is derived from a Greek word, signifying to stretch, and consists in a permanent contraction of the voluntary muscles, with irregular intervals of partial relaxation, though never complete.

There are two varieties of this disease—*idiopathic* and *traumatic*. The former proceeds from general causes, as cold, intestinal irritation, &c.; the latter, as its name signifies, is caused by external violence, and commonly results from wounds by thorns, rusty knives, nails, bayonets, &c. Tetanus has also been divided into acute and chronic. Idiopathic tetanus is in general much milder than the traumatic form, and usually yields to suitable treatment. It is traumatic tetanus that will engage our attention to-day. It is variously called, according to the particular class of muscles involved. When the muscles of the throat and jaw are affected, it is termed *trismus*, or locked-jaw; when the muscles of the abdomen, *emprosthotonos;* when those of the posterior part of the neck, and along the back, *opisthotonos;* and when the body is drawn to one side, it is styled *pleurothotonos. Trismus nascentium* is a form of tetanus attacking new-born children in hot climates, within the first nine days after birth. The earliest symptom commonly complained of in tetanus is a soreness and rigidity about the throat and jaws, as if the patient had caught cold, together with inability to rotate the head freely. After a while considerable difficulty is felt in opening the mouth, and it finally becomes impossible. A sense of tightness and constriction is now experienced at the bottom of the sternum, followed by acute pain shooting towards the spine, the muscles of which become rigidly and permanently contracted, bending the body backwards in the form of an arch, so that often it rests for support upon the occiput and heels alone. The patient is bathed in a cold perspiration and is tormented by an intolerable thirst, which cannot be alleviated, liquids being swallowed with difficulty, or the sight of them inducing violent paroxysms, causing their ejection for some distance from the mouth. In this respect the disease resembles hydrophobia, as well as in the similar effects which a draught of cold air produces, the opening of a door or window causing the most violent and frightful convulsions. There is one diagnostic sign, however, which will even in the most doubtful cases enable the surgeon to distinguish between it and hydrophobia;—in hydrophia there is always a total intermission of the spasms; in tetanus, never; partial relaxation sometimes occurs, but a complete cessation of contraction never takes place. In *opisthotonos* the muscles of the abdomen become so rigid that they are as hard as a board, and sometimes the recti muscles are ruptured by the violence of the spasms. The various muscles about the face and throat sometimes exhibit every variety and irregularity of contraction, and the countenance is so much distorted and changed that it is impossible for the most intimate friend to recognise the patient. The muscles of the eye are occasionally, though rarely affected, as well as the orbicularis palpebrarum, and levator palpebræ. The sphinter ani, and perineal muscles, are also sometimes violently and permanently contracted. When the diaphragm is thrown into spasms, we have the risus sardonicus, and it is the affection of this muscle which probably causes the pain about the ensiform cartilage. The muscles of the pharynx and larynx frequently participate in the general spasm, producing inability to swallow, and distress in breathing.

We shall speak of the effects of tetanus in the different parts of the body. The brain is rarely affected, the mind generally remaining sound, and the patient calm and collected until the last, even under large doses of opium; and this is another distinguishing feature between this disease and hydrophobia, where some mental aberration is usually observed. The bowels are singularly torpid; the skin is hot and moist; and the perspiration is said to possess a peculiar pungent odour; the pulse in general is not much affected, traumatic tetanus being not often accompanied by fever. The tongue at the commencement is moist, but afterwards becomes dry. The urine is high colored and scanty.

In tetanus, death occurs either from exhaustion or asphyxia. When from the first, it is after some days or weeks of prolonged suffering, during which time little or no nourishment has been taken; the spasms now become less frequent, the muscles relax, the pulse is very feeble, and the eye is glassy, with all the symptoms of approaching and speedy dissolution, and the patient dies worn out with suffering and pain. When death is sudden, it is produced by asphyxia. By some writers it has been erroneously attributed to spasm of the heart. That this vital organ may become affected with spasm in the course of the disease, as any other muscular structure, is possible; but there is no proof that it is ever the cause of death. It cannot be a common symptom, because generally we find the heart's action accelerated, directly opposite to the effect produced by spasm, which necessarily involves a temporary arrest in its action. The severe pain felt in the præcordial region is referrible to the spasmodic action of the diaphragm, and we must not moreover place too great reliance on the patient's account of the seat of pain, as it is very common for the lower orders to refer every uneasiness about the chest or stomach to "*the heart.*" When the patient suddenly and immediately expires, it is from asphyxia, which is produced by the sudden spasmodic closure of the glottis, or by the combined effect of irregular action in the thoracic muscles and diaphragm, preventing the expulsion of the inspired air.

Traumatic tetanus results from local injuries of various descriptions, though commonly from punctured or lacerated wounds. It follows, sometimes, amputations, castration, the operation for aneurism, &c. Severe flagellation has produced it in negroes. Baron Larrey relates a case where it was produced by the lodgment of a fish bone in the fauces. It has followed fractures and severe lacerations. It has been said that wounds of nervous and tendinous parts, are particularly liable to be succeeded by tetanus, and it would appear from credible statistics, that it most frequently results from injuries of the extremities. The magnitude or extent of the injury does not appear to exercise any influence on its production, it often succeeding the most trivial abrasions. The particular time of the invasion of tetanus after the receipt of the injury, varies from a quarter of an hour to twenty-one days. A case is related by Professor Robinson, of Edinburgh, where the patient died in fifteen minutes after having scratched his thumb with a broken china plate. Sir James Macgregor never knew it to exceed three weeks after the reception of the wound; and Sir Benjamin Brodie says, in all the cases he has seen, with a solitary exception, it manifested itself during the second week. The physician should, therefore, be careful not to pronounce his patient exempt from liability previous to the twenty-eighth day. In a case in my practice, it occurred on the eighteenth day after a gunshot injury in the hand.

Tetanus is more commonly met with in hot climates, and in warm weather, than in cold. Negroes are said to be more liable to it than whites. Females are less subject to it than males, and with them it is less fatal.

The appearances on dissection in patients who have died from tetanus, are very far from satisfactory. I have not had an opportunity of examining more than two or three cases, and the appearances corresponded with those generally given by writers. In the brain we find evident congestion, in the sinuses of the dura mater and pia mater; increased redness of the cerebral substance, and some effusion between the meninges and in the ventricles. We very often find serous effusion between the medulla spinalis and its sheath, with increased vascularity at the origin of the nerves. Injection of the substance of the spinal cord has also been noticed. Mr. Swan, in his Essay on Tetanus, observed an unusual vascularity of the cervical and semilunar ganglia of the sympathetic nerve. It has hence been concluded that tetanus is a functional nervous disorder, unattended by structural lesion, and with which inflammation has nothing to do. Mr. T. Blizard Curling, who has written an excellent Treatise on Tetanus, is of opinion that the morbid condition of the spinal marrow is confined to a particular portion of the column, the *tractus motorius*; "to which the noxious impression is communicated from distant sentient nervés at the seat of injury." It would seem to be an action independent of the vascular system. It would be most honest, however, to say that the pathology of this disease is involved in much obscurity.

We have now arrived at the most important part of our subject. Tetanus has generally been acknowledged by all writers as the most difficult to cure of all diseases. My own experience causes me to dissent from the opinion of those European surgeons who pronounce it incurable. Out of five cases that I have treated within a few years, I have been successful in three. Hennen, whose experience as a military surgeon was very great, states that he "never was so fortunate as to cure a case of acute symptomatic tetanus." He must, in the course of the campaigns in which he was engaged, have witnessed a vast number of cases. Sir James Macgregor, in his report, holds the same discouraging language. There is no disease in which so many remedies have been recommended. Indeed, I suspect the chief error has been in confiding too implicitly in one. Before detailing the plan of practice I have been led individually to adopt in this disease, I will detail the different remedies recommended at various times. The treatment is of two kinds, local and constitutional. The local treatment consists in amputation of the affected limb, and in dividing the nerves going to the seat of injury. Larrey is a strong advocate for amputation, but the testimony of the English army surgeons is unfavourable to its trial. In the very commencement of the disease, at the first manifestation of spasm, when the cause is a severe injury of the extremities, amputation may arrest the progress of tetanus; after that time it aggravates it. The division of the nerves going to the wound has been advised, and four cases are reported of its success. It should be done in the initiatory stage of the attack and before the irritation is transferred to the origin of the injured nerve.

Among the constitutional remedies, purging was, at one time, in much repute. As an adjuvant, I believe it highly useful. The bowels, as I have said before, are very costive in this complaint, and are with difficulty moved. The best cathartic I have found to be Scudamore's Mixture, consisting of Epsom salts, calcined magnesia, and wine of colchicum, it operating promptly and efficaciously. Sometimes spasm of the sphincter ani is the cause of the obstinate constipation. In such cases it must be allayed by an anodyne enema. *Mercury* has enjoyed some reputation in this disease, but the mass of testimony from the most experienced surgeons, navy, army, and others, is decidedly against it. Tetanus has been known to be developed in a patient labouring under ptyalism for some other disease. Innumerable cases are recorded where the system has been saturated with the mercury, and nevertheless the mineral apparently aggravated the intensity of the disease. We may, therefore, safely conclude that in those cases where mercury was employed, and which terminated happily, that it was a mere coincidence. *General blood-letting* I believe to be particularly injurious. Its use, in punctured and lacerated wounds, will often induce an attack of tetanus, a striking instance of which I related to you when lecturing on punctured wounds. *Local depletion* along the spine I am disposed to view favourably, as you will see hereafter. Counter-irritation in the same region, is also found to be beneficial. Of all the remedies which have been recommended in the treatment of tetanus, none has been more highly lauded than *opium*, and there can be no doubt that its efficacy is very great. Cases are recorded where the amount taken is almost incredible. In one instance, one hundred ounces of laudanum were given in the course of a month. Begin relates a case where there were administered in ten days four pounds, seven ounces, and six drachms of laudanum, together with six ounces and four drachms and a half of solid opium. The probability is, that the narcotic is not taken into the system, but remains in the intestinal canal unchanged. Abernethy found thirty drachms of undissolved opium in the stomach of a patient who died of tetanus, and he believes that the sensibility of the alimentary tube is so deadened that it sometimes ceases to be acted upon. *Tobacco* has been exhibited in tetanus, and with reputed success. *Cold affusion* was employed by Dr. Currie and Dr. Rush, and they speak highly of its effects. The *warm* and *vapour baths* have been tried, but the relief experienced was only momentary. *Tonics* and *stimulants* have been pronounced efficacious by Rush, Hosack, and others, but we have not conclusive evidence of their deserving any more confidence than a host of other remedies, equally praised at different times.

I shall close the lecture with detailing to you the plan of treatment I have myself heretofore pursued, and which has been attended with more than ordinary success. Convinced from my own observations and that of others that the seat of the disease was in the medulla spinalis, though of the precise nature of the lesion we know but little, I determined to address my remedies principally to the spine, to cup freely along the vertebral column, to use counter-irritation, to purge freely, and to exhibit anodynes to such an extent as deemed warrantable.

The precise treatment which I pursued can be best understood by detailing the following cases:

The report of the first case has been handed by Dr. Norris, who was then house surgeon of the Pennsylvania hospital. The second case by Dr. Kirkbride, who was also house surgeon in the same institution, and which was published by him in "The American Journal of Medical Sciences."

Case I.—John Kinney, aged twenty, blacksmith, was admitted into the Pennsylvania hospital, May the 3d, 1832, under Dr. Harris, for a compound dislocation of the first phalanx of the thumb. The dislocation was easily reduced, but it was found necessary to apply a splint, and bandage to the part, in order to retain it in its position. Inflammation supervening, the splints were laid aside, and the wound covered with a poultice. Pus afterwards formed at the point of injury, and extended beneath the palmar fascia, where a counter-opening was made for its discharge.

On the morning of the 17th, he was seized suddenly with pain in the back of his neck, and rigidity of jaws. The wound looked well, and discharged healthy pus; general symptoms good; bowels regular. He was cupped freely over the cervical vertebræ, and took two grains of opium. The remedies afforded almost immediate relief, and on the evening of that day all his alarming symptoms were removed.

Early on the 18th, the rigidity of the muscles returned, with occasional spasms in the neck and upper extremities. No headach, or fever; no pain or tenderness about the epigastrium. The wound looked well, and suppurated freely; cups were again applied to the cervical spine, followed by a blister, and opium gr. ii. was given, and repeated in the course of the day.

On the 19th, the symptoms mentioned continued with the addition of pain at the bottom of the sternum, extending to the spine. The muscles of the abdomen were hard and much contracted, as were also those of the neck. A blister was applied to the dorsal and lumbar spine, and a purge was given of gr. x. of calomel, and gr. viii. of ext. colocynth. comp. These medicines not operating, were followed by Epsom salts ℥ss., calcined magnesia ʒi., wine of colchicum ʒi,, and mint water ℥v. Of this draught a wine glass was given every hour, which produced evacuations, after which ʒii. of laudanum were exhibited, and produced sleep.

*May 20th.*—No pain at the back of the neck; spasms not so frequent, but general; patient speaks and swallows with less difficulty; muscles of the abdomen much contracted and hard; pain in the epigastrium; fingers of the injured hand strongly contracted, and cannot be extended. Opium gr. iii., and calomel gr. iv., were given, and to have a blister applied to that part of the spine not already sore.

*May 21st.*—Slept most of the night; general spasms continued, and were very severe when raised to have the blistered surface dressed. Bowels not moved during the night or this morning. Swallows and protrudes his tongue without difficulty. Ext. colocynth. comp. gr. viii., were administered; two hours after which a dose of castor oil was given, which operated freely. ʒjj. of laudanum was ordered every two hours until sleep was induced.

*May 22d.*—Slept but little; no desire for food; complains of pain at the bottom of the sternum. Muscles of the injured hand continue strongly contracted; general spasms severe. Bowels twice moved during the night. As the blistered surface on the back was nearly dry, the remedy was re-applied along the whole spine. Tincture of opium ʒjjj., were given every two hours.

*May 23d.*—Body is to-day thrown more backwards than it has been heretofore. Other symptoms the same. Bowels were open during the night. Laudanum continued in the same dose every two hours.

*May 30th.*—There has been no material change since the last report, until to-day, when he seems rather worse. Spasms come on more frequently, and now affect the muscles of the lower, more than the upper extremities. Since the 23d there has been a great want of sleep, and his sufferings have been extreme. The warm bath has been tried without relief. The laudanum is continued in same dose, and at the same intervals, when awake, and the bowels kept freely open by the colchicum mixture, no evidence of narcotism being produced by the laudanum. Blister kept sore; a grain of acet. morph. to the blistered surface three times a day.

*June 1st.*—Diet has consisted of gruels and chicken water. To-day his appetite has failed, and and is much more debilitated. Sago, with wine in it, was given freely, and was grateful. Spine was kept sore. Very little discharge from the wound, which is without pain, and the head of the bone is now covered by healthy granulations; poultice continued; laudanum as usual.

*June 6th.*—On the 4th instant, a blister was re-applied to the whole spine; the acetate of morphia as before to the blistered surface. Complains now of no pain. Bowels kept free by the colchicum mixture, which always operates kindly and freely. Laudanum continued as before.

*June 10th.*—Mind seems to-day much affected; bursts into tears when spoken to, and can give no reasons for so doing. This has been the case in a slight degree since the commencement of disease. The abdomen still much contracted. Sleeps more at night. Blister again re-applied to the whole length of the spine. Appetite good; appearance of the wound the same. Lessened the quantity of laudanum to ʒjj. every two hours when awake. Nourishing soups, and sago with wine in it.

*June 13th.*—Improves slowly. Has had no spasms for the past two days. Mind tranquil; abdomen softer; wound looks well, and is cicatrizing. Blister kept sore, and laudanum reduced to ʒj. every three hours.

*June 20th.*—Is to-day free from tetanic symptoms, abdomen being soft and natural. Laudanum has been gradually diminished in quantity, and is to-day omitted. Blister has been allowed to heal. Full diet. The wound of the thumb is nearly cicatrized.

Case II.—A coloured woman, whilst wiping an old rusty knife, about 2, P. M., on the 8th June, 1834, cut the end of the index finger of the left hand. It was a superficial wound, about three-fourths of an inch long. Early on the morning of the 10th, she was attacked with symptoms of tetanus. Dr. Harris saw her at 1, P. M., of that day. He found that she had taken sixty drops of laudanum. He ordered the dose to be immediately repeated. At this time there was much excitement of mind, approaching to delirium; cephalalgia; anxious expression of countenance; a frequent and weak pulse, which, however, from the violence of the spasms, could not be accurately counted. The flexor muscles of the upper extremities, those of the abdomen, and several of the face, contracted. The jaws were stiff. There was acute pain in the left side, and at the bottom of the sternum. No spasms of the lower extremities. In this condition she entered the hospital at 4 o'clock, P. M. She was ordered tinct. opii gtt. xl., to be repeated every half hour. Whole extent of spine to be covered with sinapisms. By 6, P. M., she had taken two hundred and sixty drops of laudanum. The spasms were now more frequent and violent, and nearly constant. The inferior extremities were now as much affected as the superior. Pain excrutiating. f℥xviij. of blood were taken by means of cups along the spine. A blister was applied extending from the occiput to the sacrum. Her screams from the pain were now so great, that it was found necessary to remove her to a separate ward. The delirium increased, and the spasms became so violent, that several attendants were requisite to keep her in bed. She was so restless that it was impossible to keep the blister on the spine. The lin. canthar. was in place rubbed briskly in, whenever an opportunity occurred. One hundred drops of laudanum were administered at 6 o'clock, P. M. Calomel gr. vj., morph. sulph. gr. ij., at 7, and morph. sulph. gr. ii., at 8, P. M. Ordered an enema, containing six hundred drops of laudanum, at 9½ o'clock. In a quarter of an hour afterwards the spasms became less violent, and speedily ceased entirely. 10½, P. M., evidently narcotized. Sleep deep; respiration four; breathing stertorous; pinching incommoded her, yet when roused at all she instantly relapsed into insensibility. Stimulating enema ordered. f℥vjjj. of blood were taken from the head by cups; mustard foot-bath. She afterwards seemed rational, and slept well for the rest of the night. Blister re-applied to the spine.

*June 11th*, 6, P. M.—No spasms since last report. When roused, talks rationally; "feels stupid;" has headach; some rigidity of jaws; tongue white and clammy; pulse one hundred and twelve, and soft; bowels not open since the attack; wounded finger not painful—poulticed. Ordered f℥iij. of Scudamore's mixture; same quantity repeated in two hours.

10, P. M.—Bowels freely purged. Patient was doing well until 4, P. M., when, becoming excited by the visits of her friends, there was a return of the spasms, pain in the side, and scrobiculus cordis. She took one hundred and sixty drops of laudanum by the mouth, and two hundred were administered by an enema. This relieved the symptoms until 8½, P. M., when they again returned. Two doses of laudanum of eighty drops each were administered, and an enema of two hundred, but without effect.

*June 12th*, 10, P. M.—She had a return of the spasms since the last report, by the severity of which the quantity of laudanum was regulated. Bowels moved twice during the night. The amount of opium taken during the last twenty-four hours has been five hundred and fifty drops by the mouth, and six hundred by the rectum.

*June 13th*, 10, P. M.—Slept greater part of the last night; has since had some returns of her former symptoms; pulse quite soft, and irregular; tongue slightly furred; rational. Since last report, with two intermissions, she has taken one hundred drops of laudanum every two hours, and four enemata containing three hundred drops each.

*June 14th, night.*—Slight return of spasms at 8, A. M., which were relieved by an enema of three hundred drops of laudanum, in addition to which she took one hundred drops every two hours by the mouth; ordered Scudamore's mixture as before. Tinct. opii., gtt. c., q. 4th h.

*June 15th.*—The patient has had several free discharges from the bowels; spasms less frequent and violent; pulse weaker and intelligence dull; pain continues at lower extremity of sternum. Morph. sulph. gr. iv., have been sprinkled on the blistered surface over spine.

*June 17th.*—Morph. sulph. gr. iv., to blister, with one enema of three hundred drops of laudanum, with one hundred by the mouth every two hours, continued yesterday. To-day same dose, and morph. sulph. gr. iv., twice a day to blister. The purging mixture ordered, and the laudanum continued as before.

*June 18th.*—Much better; no tendency to spasm; intelligence good; countenance animated; appetite good; pulse ninety; no pain in spine, or epigastrium; bowels well purged; she takes tinct. opii., gtt. c., every four hours.

*June 21st.*—The opiate has been gradually diminished, so that to-day she will have taken but five doses of forty drops each. Complained to-day of pain in the injured finger which was relieved by the application of a grain of sulphate of morphine to the wound; purging mixture repeated.

*June 24th.*—The patient's tongue coated; little appetite; no pain; sleeps well. She takes mass. hydrarg. gr. iii. twice a day; a Seidlitz powder in the morning; tinct. opii. sixty drops at bed time.

On the first of July she was discharged, cured.

Case III.—Mary W——, aged twenty-seven, wounded the palm of her hand with a piece of broken china, on the 25th of August, 1834.

On the fourth day after the wound was inflicted, she was seized with pain in the cervical spine, hoarsenes, and stiffness in the muscles of the face, which she attributed to cold, not apprehending that these symptoms had any connection with her injured hand.

Dr. Hewson, the family physician, was sent for, who at once perceiving the nature of her disease, sent for me to consult on the case. At the time we met, her jaws were fixed, the muscles of the back were violently contracted, with the usual pain at the bottom of the sternum. The plan of treatment pursued in the former cases was adopted in this and with complete success. The violent symptoms were not subdued until the fifth day after the attack. The symptoms were not less violent than in the other cases. A want of time will prevent me going into further detail.

Case IV.—In October, 1835, Mr. W——, aged eighteen years, in crossing a fence, his gun accidentally exploded, and discharged a large load of shot through his left wrist. The integuments and tendons were lacerated, and the carpal bones fractured. His suffering after the first four days was not severe. The wound suppurated freely, exhibited a healthy aspect, and all his functions were perfectly performed. On the thirteenth day after the accident, symptoms of tetanus supervened, but at first mild in its form; much less so than any of the preceding cases.

From past experience I had sanguine hopes that I should succeed in controlling the disease. My anticipations were not realized. The patient died on the fifth day of the attack, though I pursued the same course of treatment as in the cases which terminated favourably.

Case V.—In June, 1836, ———, was admitted into the Pennsylvania hospital with a lacerated wound of the arm, occasioned by an explosion of gunpowder while blowing rocks. On the ninth day after the infliction of the injury, he was attacked with trismus, followed the next day by general tetanic symptoms.

In this case, also, the same treatment was pursued, but without success. The patient died on the fourth day.

In the three patients in which this formidable desease terminated favourably, they suffered no narcotism, nor inconvenience from the quantity of laudanum taken. In the two fatal cases the patients became narcotized before they had taken one fifth of the quantity exhibited to the others. Such was their idiosyncracy that they could not take a sufficiency of anodyne to control the spasms, while the cups, blisters, &c., were acting on the spine, which is thought to be the true seat of the disease.

